# Prognostic and Immune Infiltration Value of Proteasome Assembly Chaperone (PSMG) Family Genes in Lung Adenocarcinoma

**DOI:** 10.7150/ijms.78590

**Published:** 2023-01-01

**Authors:** Do Thi Minh Xuan, I-Jeng Yeh, Che-Yu Su, Hsin-Liang Liu, Hoang Dang Khoa Ta, Gangga Anuraga, Chung-Chieh Chiao, Chih-Yang Wang, Meng-Chi Yen

**Affiliations:** 1 Graduate Institute of Cancer Biology and Drug Discovery, College of Medical Science and Technology, Taipei Medical University, Taipei 11031, Taiwan.; 2 Department of Emergency Medicine, Kaohsiung Medical University Hospital, Kaohsiung Medical University, Kaohsiung 80708, Taiwan.; 3 Graduate Institute of Clinical Medicine, College of Medicine, Kaohsiung Medical University, Kaohsiung 80708, Taiwan.; 4 Ph.D. Program for Cancer Molecular Biology and Drug Discovery, College of Medical Science, Taipei Medical University, Taipei 11031, Taiwan.; 5 Department of Statistics, Faculty of Science and Technology, PGRI Adi Buana University, East Java, Surabaya 60234, Indonesia.; 6 TMU Research Center of Cancer Translational Medicine, Taipei Medical University, Taipei 11031, Taiwan.

**Keywords:** *PSMG* family genes, Lung cancer, HSP90/PI3K/Wnt

## Abstract

The complexity of lung adenocarcinoma (LUAD) including many interacting biological processes makes it difficult to find therapeutic biomarkers for treatment. Previous studies demonstrated that *PSMG* (proteasome assembly chaperone) family members regulate the degradation of abnormal proteins. However, transcript expressions of this gene family in LUAD still need to be more fully investigated. Therefore, we used a holistic bioinformatics approach to explore *PSMG* genes involved in LUAD patients by integrating several high-throughput databases and tools including The Cancer Genome Atlas (TCGA), and Kaplan-Meier plotter database. These data demonstrated that *PSMG3* and *PSMG4* were expressed at significantly higher levels in neoplastic cells than in normal lung tissues. Notably, increased expressions of these proteins were correlated with poor prognoses of lung cancer patients, which probably confirmed their fundamental roles in the staging of LUAD tumors. Meanwhile, it was also indicated that there were positive correlations between *PSMG* family genes and the immune response, metabolism of ubiquinone, cell cycle regulatory pathways, and heat shock protein 90 (HSP90)/phosphatidylinositol 3-kinase (PI3K)/Wnt signaling. Experimental data also confirmed that the knockdown of *PSMG4* in LUAD cell lines decreased cell proliferation and influenced expressions of downstream molecules. Collectively, this study revealed that *PSMG* family members are novel prognostic biomarkers for LUAD progression, which also provide new therapeutic targets of LUAD patients.

## Introduction

As the most fatal malignancy worldwide, primary lung cancer generally ranks first in both incidence and mortality of cancers. Recent statistics show that fatal cases of lung cancer consistently accounted for approximately one-fourth of all cancer deaths, almost double the number of fatal cases caused by second-place colorectal cancer 1. According to the traditional classification, lung cancer is broadly grouped into two main types: small cell lung cancer (SCLC) - the aggressive one associated with previous smoking that makes up to 15% of total cases - and non-SCLC (NSCLC) - the less aggressive but more-prevalent one that accounts for the majority (up to 85%) of the remainder. Both of them are characterized by distinct cell morphology and topology which lead to significant differences in tailored treatments and diverse disease prognoses 2. Among the three main subtypes of NSCLC which can be distinguished by distinct histopathological characteristics, lung adenocarcinoma (LUAD) appears to be the most common type of primary lung cancer found in lifelong non-smokers and individuals with a history of smoking as well. Despite recent advances in diagnostic approaches, including imaging-based screening tests, sputum cytology, and modern biopsy techniques, up to 75% of patients are detected in later stages, when metastases are present. Tracking numbers provided by the Surveillance, Epidemiology, and End Results (SEER) database reveal that prognoses of lung cancer are far more troubling than other leading cancer sites (colon, breast, and prostate), with overall 5-year relative survival peaking at 26% but plummets to 8% or even lower in cases of distant metastases. In contrast, patients diagnosed at early stages with localized tumors not only benefit from surgical resection but also from multiple choices of treatment options, with cure rates of as high as 64%. Therefore, early detection of lung cancer in asymptomatic patients is of utmost concern in an effort to improve the disease's dismal outcomes.

Recent genomic studies have confirmed the presence of tumor-harboring somatic mutations and alterations of specific genes in individuals with LUAD, most notably mutation of the epidermal growth factor receptor (*EGFR*) and anaplastic lymphoma kinase (*ALK*), which paved the way for novel classes of drugs known as targeted kinase inhibitors. In addition, activating mutations in certain genes such as *KRAS, BRAF, ERBB2*, and *PIK3CA*; translocations found in *RET, ROS1*, and *ALK*; and fusions detected in the *NRTK1/2/3* genes have been target subjects of a significant number of on-going clinical trials 3, 4. However, specific types of mutation, such as loss-of-function mutations and deletions in tumor suppressor genes, have yet to be therapeutically exploited 5-7. Therefore, extensive knowledge of carcinogenesis driver gene alterations will become even more critical to guide the clinical care of LUAD patients. In an attempt to extend current knowledge of details of how molecular signaling is involved in this malignant disease, this study was designed to seek novel biomarkers that can contribute to early detection and prognostic evaluation of LUAD.

The 26S proteasome is a large ATP-dependent protease complex built from a 20S catalytic core particle (CP) responsible for protein degradation and one or two 19S regulatory particles (RPs) essential for ubiquitin recognition. The 20S CP is a combination of two outer α-rings and two inner β-rings stacked in a given order of αββα, each composed of seven homologous subunits respectively referred to as α1-α7 and β1-β7 8. Apart from the most prominent role in clearing malfunctioned and damaged proteins, recent studies further elucidated how the human ubiquitin-proteasome system takes control of cell-cycle progression, cellular survival, apoptosis, and activation of nuclear factor (NF)-κB, all of which are emerging hallmarks of cancer accelerated by abnormal proteolytic activity in a genetic mutation-independent context 9-13. To date, three proteasome inhibitors have been brought to clinical use to treat myelomas and leukemia 14. As an organelle made up of multi-subunits, proteasomes lack the ability to spontaneously assemble 15, 16. Initiation and proper formation to create complete and biologically active proteasomes are thus guided and monitored by proteasome assembly chaperones (PSMGs), which are dedicated to preventing aberrant dimerization and ensuring proper incorporation among subcomplexes 16-20. Given that proteasomes are crucial for intracellular protein degradation and turnover, disorders of proteasome assembly are frequently linked to cellular dysfunction and diminished abilities to respond to proteotoxic stresses, and these disorders have also been well-documented during the past few decades 21. Being part of the human chaperone complex, four members of the proteasome assembly chaperone family genes, referred to as *PSMG1~4*, were previously reported to be associated with multiple disorders 22-25. More specifically in terms of malignancies, earlier studies revealed that *PSMG1* was related to increasing susceptibility to inflammatory bowel disease which can lead to colon cancer-related diseases 26, 27, whereas a co-expression relationship of *NUP37* with *PSMG1* was proposed to play a specific role in breast cancer 28. *PSMG3* is characterized as an oncogenic driver factor in various types of cancer, remarkably breast cancer and cervical squamous cell carcinoma 29. *PSMG4* variants, on the other hand, were some of the differential proteomics identified in Epstein-Barr virus-associated gastric cancer. Since relationships between the *PSMG* gene family and cancers have barely been described thus far, our study aimed to better elucidate roles of the *PSMG* gene family in LUAD using multi-approach bioinformatics analyses.

By leveraging high-throughput screening analyses performed in public databases, we previously reported that certain family genes encoded for proteasome complexes are associated with poor prognoses and progressive proliferation of various cancer types 30-32. In this study, differential expression analysis of *PSMG* family members was first examined at the transcriptome level in a LUAD cohort of The Cancer Genome Atlas (TCGA), and later confirmed at the proteome level by immunohistochemical (IHC) staining of LUAD specimens. The survival significance of each *PSMG* gene was evaluated through corresponding estimated Kaplan-Meier curves of overall survival (OS). Next, the significance of *PSMG* family genes were investigated through functional enrichment analyses, by which coexpressed gene interaction networks, relevant biological processes, and functional annotations were revealed. Additionally, extensive analyses were also performed to clarify the family's relationships with immune infiltration levels. Finally, wet lab validation was conducted to confirm the roles of the most significant *PSMG* genes in relation to LUAD tumor proliferation and their influence on downstream signaling pathways.

## Materials and Methods

### Cell culture and RT-qPCR

The human lung alveolar type II epithelial cell line (A549), kindly gifted of Prof. Chiou-Feng Lin from Taipei Medical University (TMU; Taipei, Taiwan) 33. Cells were cultured in Dulbecco's modified Eagle medium (DMEM) (90-113-PB, Corning, USA) supplemented with 10% fetal bovine serum (Avantor, USA) plus 1% penicillin/streptomycin (Corning), and maintained at 37 °C in a humidified incubator in 5% CO_2_. *PSMG4* gene silencing was generated using a small hairpin (sh)RNA knockdown vector system and lipofectamine 2000 (Life Technologies Inc., Carlsbad, CA) transfection was carried out according to the manufacturer's protocol as we previously described 34, 35. All shRNA vectors harboring puromycin and enhanced green fluorescent proteins, including two human PSMG4 shRNA, and a non-target control (pLKO.1) shRNA against luciferase (shLuc), were constructed by the National RNAi Core Facility (Academia Sinica, Taiwan; https://rnai.genmed.sinica.edu.tw). Stable clones expressing pLKO_TRC005 were selected by constant treatment with puromycin (2 µg/mL) from 72 hours lipofectamine transfection. A non-target control (pLKO.1) shRNA against luciferase (shLuc) was employed as an expression control. On day 28 post-transfection, the efficacy of gene silencing was further confirmed by comparing quantitative reverse-transcription polymerase chain reaction (RT-qPCR) of two groups of shPSMG4-transfected A549 cells versus pLKO.1(vector control)-transfected cells. Total RNAs from stable clones of the A549 cell line were extracted using the GENEzol™ TriRNA Pure Kit (Geneaid Biotech, Taiwan) following the manufacturer's protocol, whereas complementary (c)DNA was subsequently reverse-transcribed using a PrimeScript Synthesis Kit (Takara Bio, Japan). An RT-qPCR was performed using cDNAs as templates and GoalBio SYBR green master mix (Hycell International, Taiwan) on the Roche Light Cycler 96 platform. Primer pair sequences targeting *PSMG4*, *HSP90AA1*, and 18S ribosomal (r)RNA were constructed by MDBio (MDBio, Taiwan). Relative fold changes in expression of the *PSMG4* and *HSP90AA1* genes were calculated by the delta-delta Ct (2^-∆∆Ct^) method after being normalized against the Ct value of *18S* rRNA as the housekeeping gene. All experiments were performed in triplicate. RT-qPCR results are presented as the mean ± standard deviation 36-38.

### Colony-formation assay

A549 cells of three experimental groups were seeded at the same density (1000 cells/well) in six-well plates for 2~3 weeks until macroscopic colonies had formed. After that, the medium was discarded, and cells underwent absolute methanol fixation in 20 min at room temperature, followed by short-term incubation with 2% methylene blue staining in 30 min at room temperature. The number of colonies formed in each well was counted under a low-magnification (×100) light microscope. Experiments were performed in triplicate. Results were represented as the mean ± SD of triplicate data 39-41.

### UALCAN, Cancer Cell Line Encyclopedia (CCLE), and Kaplan-Meier analysis

TCGA database is the largest and most widely used public resource providing gene expression profiling, gene methylation profiling, copy number variation profiling, single-nucleotide polymorphism (SNP) genotyping, genome-wide DNA methylation profiling, microRNA profiling, and exon sequencing. Among the many computational tools that have been developed to assist scientists in performing specific analyses based on TCGA database, UALCAN (http://ualcan.path.uab.edu) is an interactive web tool capable of calculating the relative expression of a queried gene across a specific tumor or various tumor sub-groups against normal samples. Relevant analyses are based on either individual cancer stages, tumor grades, or other clinicopathological features. In our study, we examined alterations in terms of the transcriptome of four *PSMG* family genes in 515 primary LUAD samples versus 59 normal samples, along with different clinicopathological characteristics and stages. Next, we used CCLE to further explore the gene expression level of all *PSMG* members on a broader scale 42. This web-based application gives users access to more than 1000 human cancer cell lines. Additionally, independent LUAD cancer cell lines were subjected to the integrated RNA-Seq Aligned Reads method to plot the corresponding expression of each *PSMG* gene. The KMplot survival database (https://kmplot.com/) was leveraged to explore which *PSMG* family members are novel prognostic biomarkers 43. The comparisons between two groups of patients were performed with 95% confidence intervals (CIs) of the hazard ratio (HR) and fixed log-rank p-value as we previously described 44-46.

### Tumor Immunological Estimation Resource (TIMER) database analysis

The latest version (2.0) of TIMER, available at http://timer.cistrome.org/, is generally acknowledged as an efficient tool for conducting systematic analyses of host immune infiltrates among various cancer types and associated diseases 47. In other words, using the DiffExp module and default parameters, this webserver assists in estimating the abundances of six immune cell types grouped into two distinct lineages in the tumor microenvironment (TME): B cells, cluster of differentiation-positive (CD4^+^) T cells, and CD8^+^ T cells that belong to lymphoid lineage cells, together with neutrophils, macrophages, and dendritic cells (DCs) that belong to myeloid lineage cells. Finally, scatterplots were employed to show the associations, with the x-axis representing *PSMG* gene expression levels and the y-axis showing markers for immune cells that infiltrate lung tumors.

### Functional enrichment analysis of *PSMG* target genes

The InteractiVenn tool was selected to generate a one-way Venn diagram that presents the overlap and number of genes linked with expressions of *PSMG* target genes across two given datasets obtained from TCGA databases (available at the cBioPortal platform). This online web tool (available at http://www.bioinformatics.com.cn/srplot), together with the MetaCore platform to investigate the potential pathways and involved networks, as well as an online platform (http://www.bioinformatics.com.cn/ en?keywords=heatmap) was conducted for data visualization. A *p*-value of <0.05 was set as the cutoff value 48-50.

## Results

### *PSMG* family members play important roles in developmental stages of LUAD

**Figure 1:**
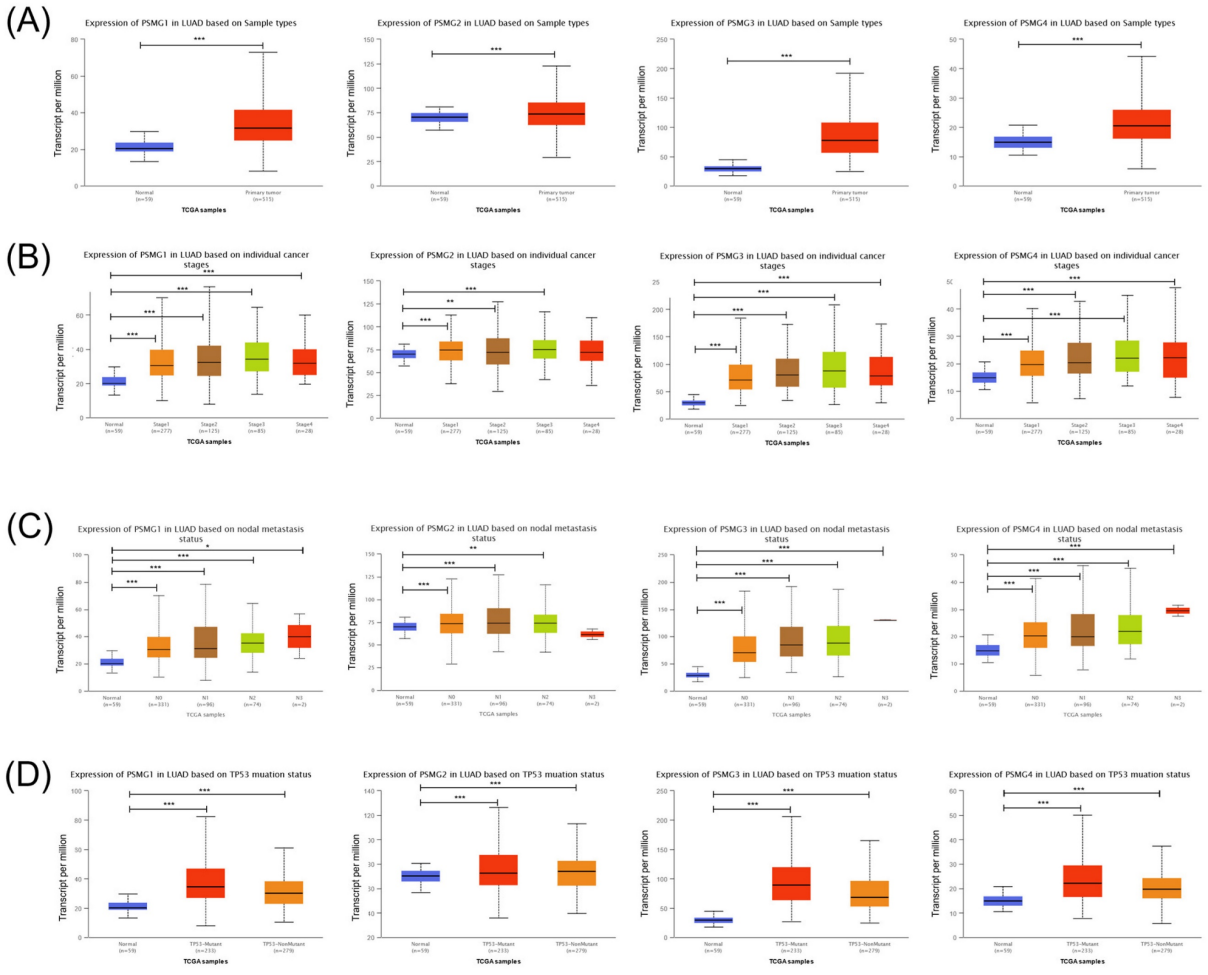
** Transcriptional expression of PSMG (proteasome assembly chaperone) family members in lung adenocarcinoma (LUAD). (A)** Boxplot of PSMG transcriptomic levels recorded in LUAD patients (red) compared to healthy individuals (blue). Box plots of variations in transcriptomic levels of PSMG1~4 recorded in normal individuals and LUAD patients **(B)** from stages 1 to 4 **(C)** grouped into four subgroups (N0~N3) based on the regional lymph node metastasis status **(D)** with and without the *TP53* mutation. A *t*-test was applied considering *p*<0.05 as a significant difference.

Prior studies discovered six *PSMG* family members in *Homo* species as well as the significant roles in cancer progression of some of them. We wanted to further identify *PSMG* family gene signatures in relationship with LUAD development. Results demonstrated that compared to normal tissues, all *PSMG* family genes had high expression levels in LUAD tissues. Surprisingly, we found that *PSMG4* had positive correlations with the tumor stage, especially with the highest expression in stage 4 LUAD. *PSMG1, PSMG3,* and *PSMG4* had positive correlations with the nodal metastasis status. In addition, since the *TP53* mutation was correlated with LUAD development, we also found that *PSMG1, PSMG3,* and *PSMG4* had the highest expression in *TP53* mutant LUAD. It was suggested that *PSMG* family member overexpression may promote tumor growth, and overexpression of *PSMG1, PSMG3,* and *PSMG4* may further promote the development of cancer metastasis. Therefore, we decided to perform further bioinformatics analyses on these family genes in LUAD (Figure 1).

### Associations of *PSMG* family gene interpretations in cancer cell lines with clinicopathological parameters of LUAD

**Figure 2:**
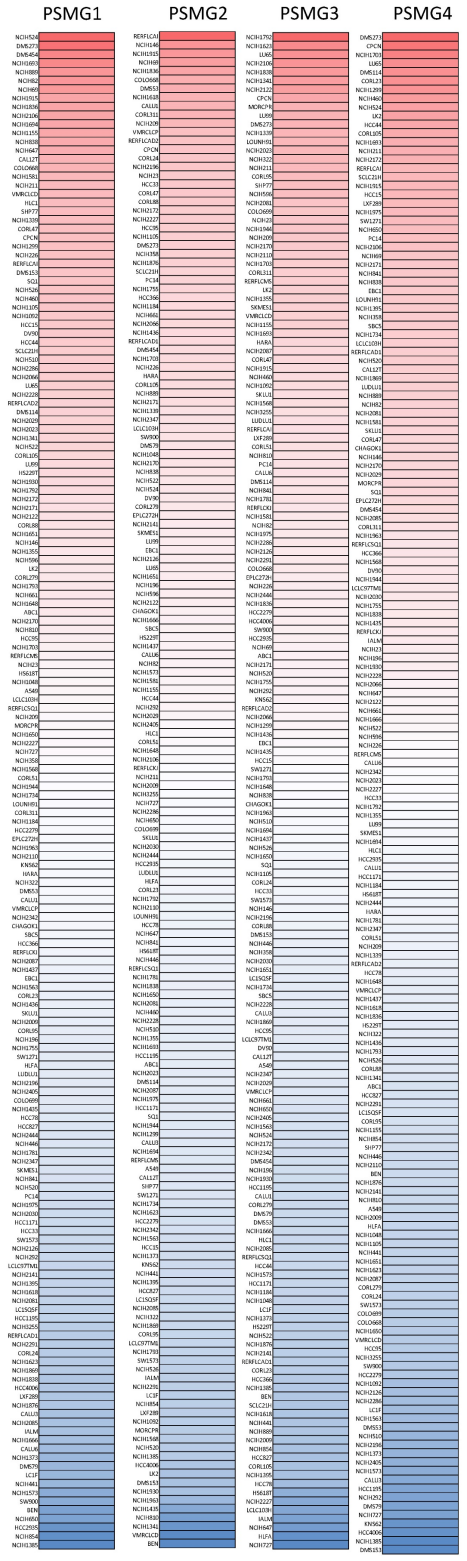
** Expression degree of PSMG (proteasome assembly chaperone) family members among common types of lung adenocarcinoma (LUAD) cell lines in a heatmap.** Varying degrees of upregulated expressions (red color), downregulated expressions (blue color), and no changes in expression of *PSMG1~4*, measured in different LUAD cell lines.

After properly examining differences in *PSMG* family gene expressions between neoplastic and normal tissues, we next performed a comprehensive analysis of *PSMG* family genes in lung cancer cell lines via the CCLE dataset. These data also indicated that *PSMGs* were overexpressed at the messenger (m)RNA level measured in the lung cancer cell lines (Figure 2).

### Relationships between disease prognostication and *PSMG* gene expression levels measured in tumor specimens and survival statuses

**Figure 3:**
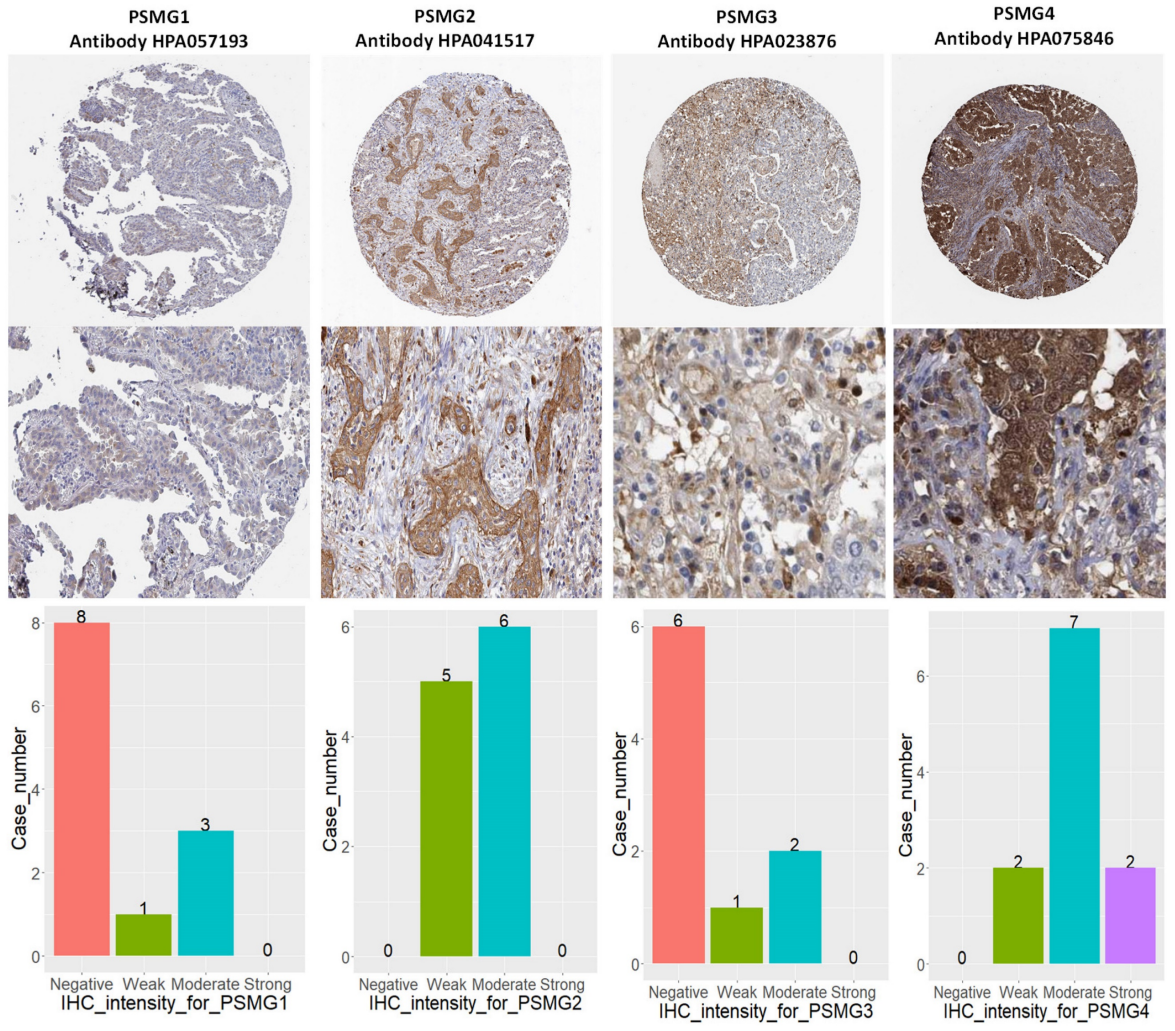
** IHC intensity patterns of PSMG1~4 (proteasome assembly chaperone1~4) recorded in lung adenocarcinoma (LUAD) patient tissues. (A)** PSMG1~4 protein expressions of LUAD tissue staining patterns, along with corresponding healthy tissues. **(B)** Bar chart of case numbers grouped according to the level of staining intensity for PSMG1~4 in LUAD tissues.

**Figures 4:**
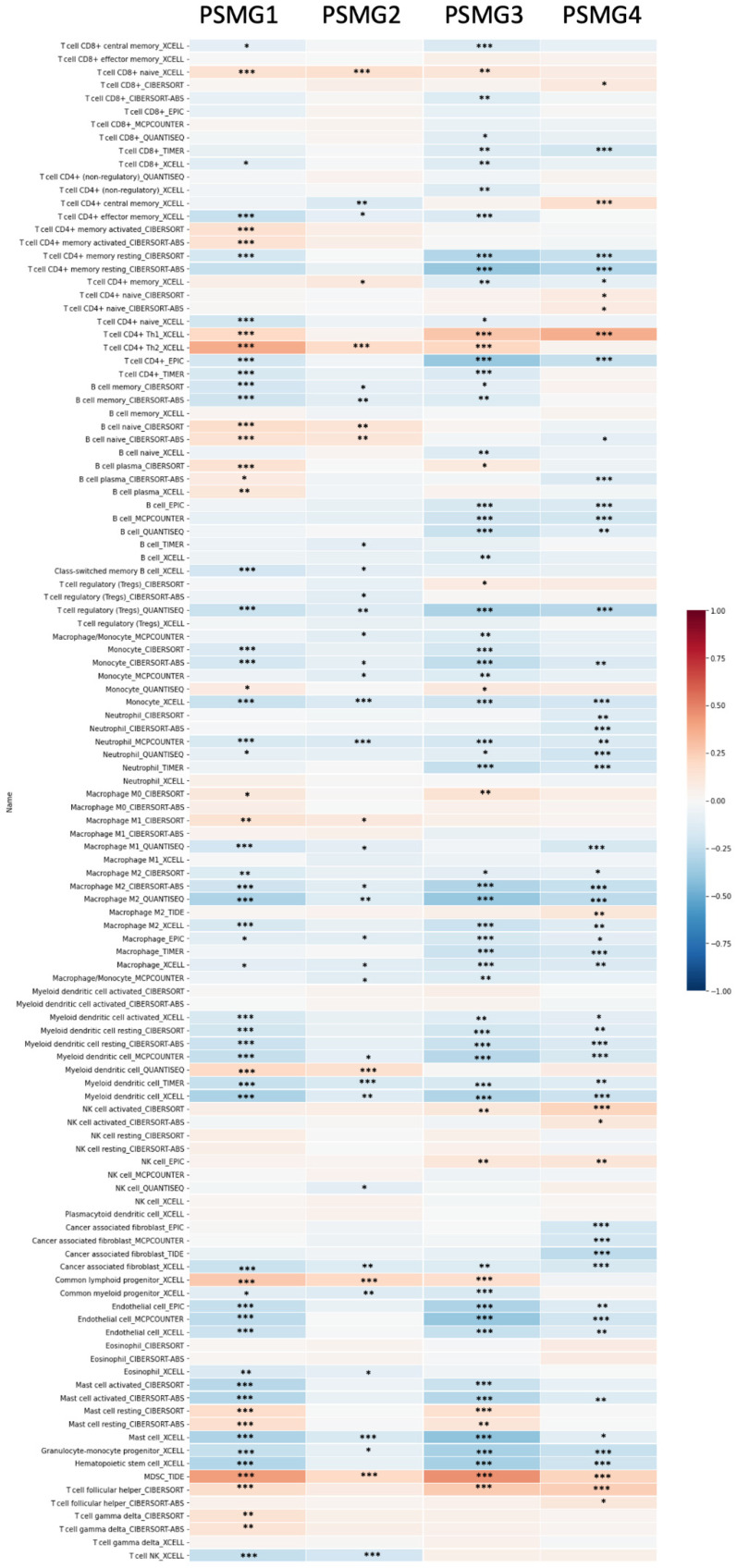
** Correlations between gene expressions of *PSMG1~4* (proteasome assembly chaperone 1~4) and immune infiltration levels among lung adenocarcinoma (LUAD) patients in TCGA cohorts through data mining from the TIMER database.** Using the "Purity Adjustment" option of TIMER, correlations between expression levels of six major immune cell populations and four subtypes of breast cancer were evaluated based on seven cell type quantification algorithms (xCell, CIBERSORT, CIBERSORT abs. mode, EPIC, MCP-counter, TIMER, and quanTIseq). Results are presented as a Pearson correlation coefficient (*r*) that ranges from -1 (negative correlation: blue) to +1 (positive correlation: red). Three statistical significance levels were reported, including * *p* < 0.05, ** *p* < 0.01, and *** *p*< 0.001.

**Figure 5:**
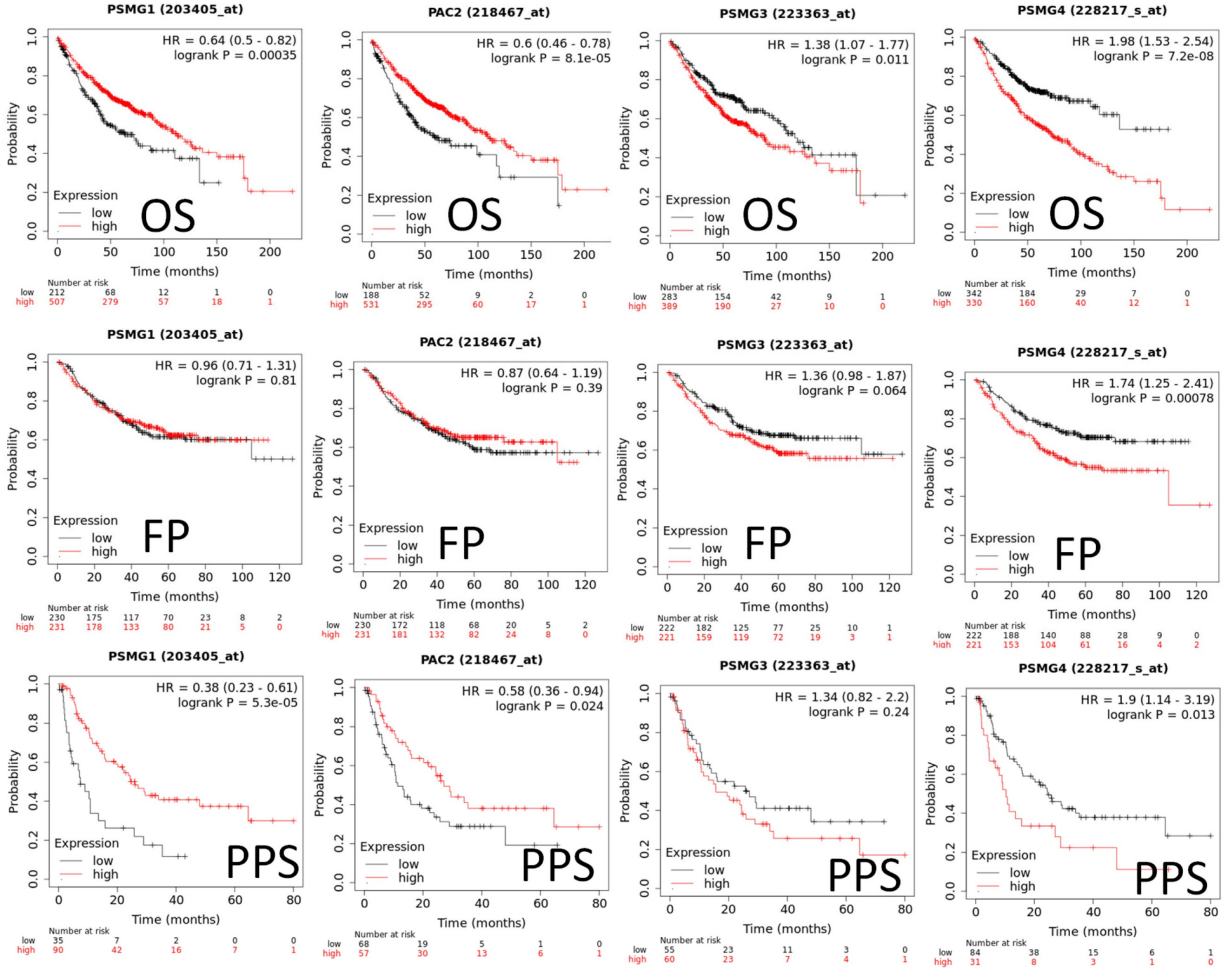
**Predicted survival analyses according to expression levels of PSMG1~4 (proteasome assembly chaperone 1~4) in lung adenocarcinoma (LUAD) patients.** The two survival curves respectively present percentages of LUAD survivors over time, in patients with low (black curves) and high (red curves) expressions of *PSMG1~4*. Elevated mRNA levels of the *PSMG1* and *PSMG2* genes resulted in good prognoses, whereas elevated mRNA levels of *PSMG3* and *PSMG4* showed the contrary. (HR, hazard ratio; OS, overall survival; FP, first progression; PPS, post-progression survival; *p* < 0.05 was considered statistically significant).

Since samples from LUAD patients included different expressions of *PSMG* family members, we further explore how these target genes take part in particular aspects of tumor progression prior to investigating the clinical relevance. Hence, the intensities of antibodies indicated in clinical LUAD specimens were extracted from the Human Protein Atlas database. IHC images revealed densely and moderately distributed *PSMG* family protein expressions in LUAD samples (Figure 3). In addition, when we performed a required analysis on the TIMER database, *PSMG* member genes also indicated relevance to the immune infiltration profiles of LUAD, which included six various tumor-infiltrating immune cell types grouped into two separate groups: B cells, CD4^+^ T cells, and CD8^+^ T cells that belong to lymphoid lineage cells, together with neutrophils, macrophages, and DCs that belong to myeloid lineage cells (Figure 4). The KM plotter database also indicated that most *PSMG* members were associated with different LUAD survival statuses. High expressions of *PSMG1* and *PSMG2* in LUAD were correlated with good OS, FP, and PPS. In contrast, high expressions of *PSMG3* and *PSMG4* were correlated with poor OS, FP, and PPS (Figure 5). These survival data imply that *PSMG3* and *PSMG4* have oncogenic roles in LUAD progression (Supplementary Figure S1).

### Pathway and network analysis of *PSMG* family member genes

**Figure 6:**
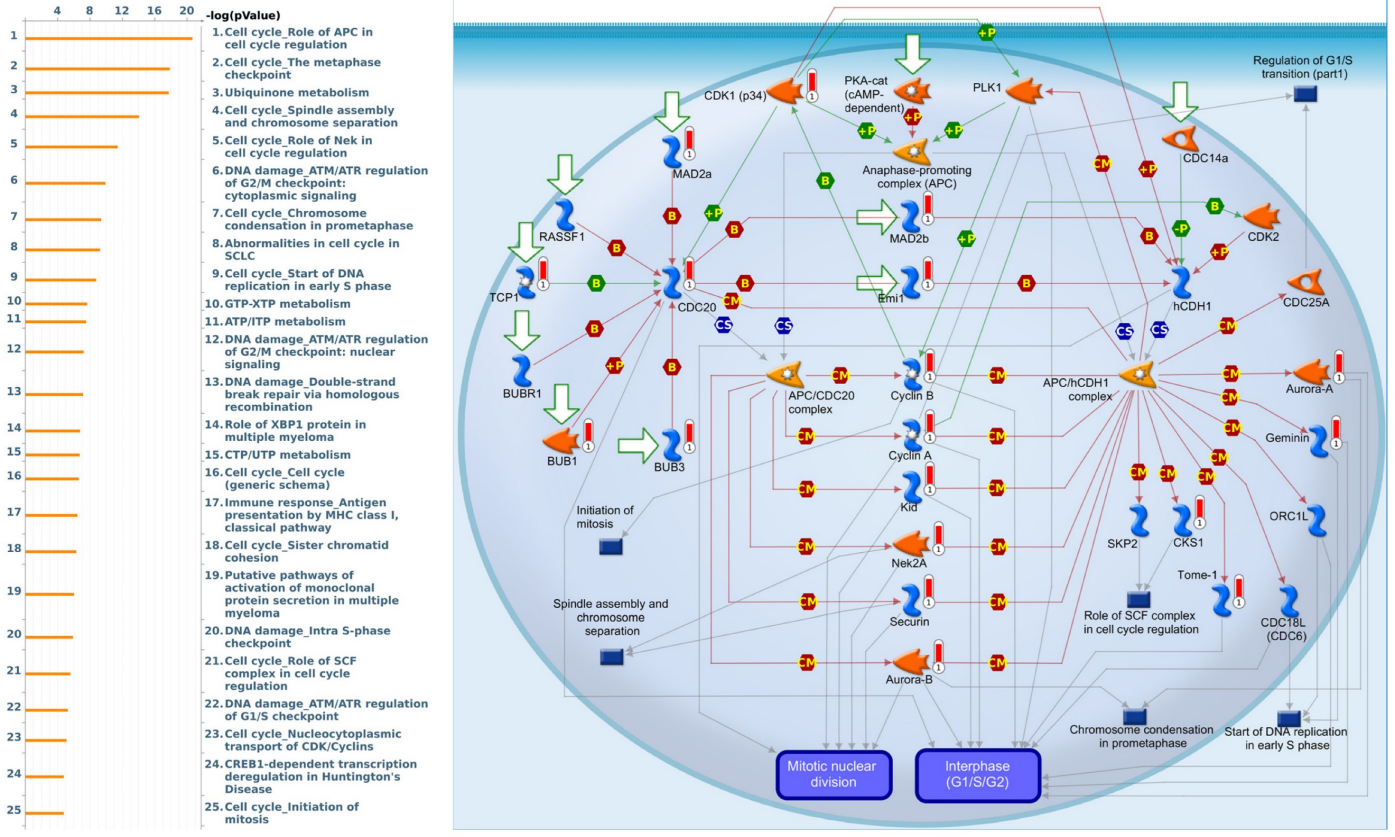
** Significantly associated signaling pathways regulated by top genes coexpressed with *PSMG1* (proteasome assembly chaperone 1), as predicted by MetaCore.** Top 10% coexpressed genes with *PSMG1* shared from TCGA databases were selected for gene expression network predictions using "biological processes" of MetaCore. The involved pathways were ranked in order of decreasing -log[*p* values]. Regulatory pathways for the cell cycle are among the most significant ones.

**Figure 7:**
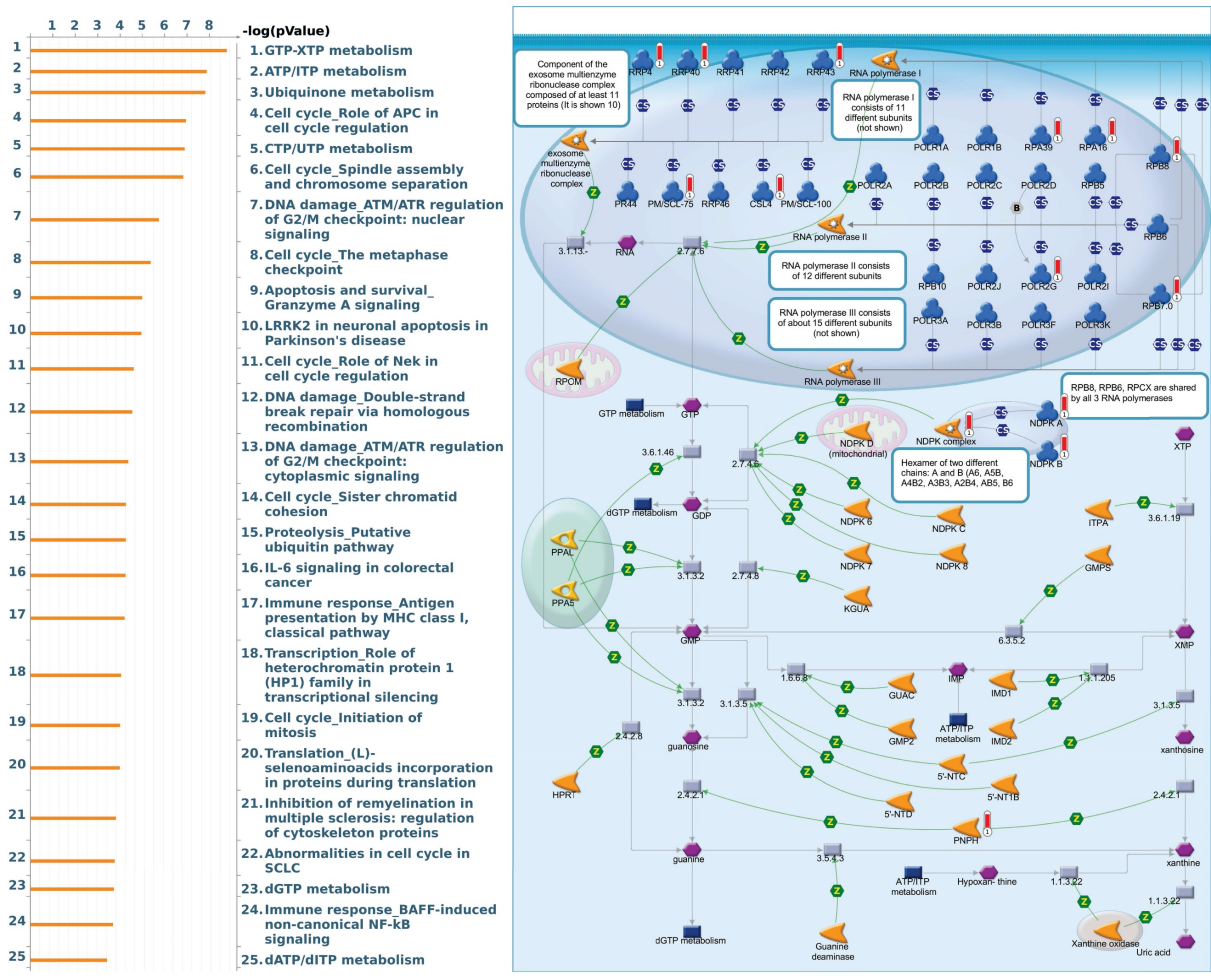
** Significantly associated signaling pathways regulated by top genes coexpressed with *PSMG2* (proteasome assembly chaperone 2), as predicted by MetaCore.** The top 10% coexpressed genes with *PSMG2* shared from TCGA databases were selected for gene expression network predictions using "biological processes" of MetaCore. The involved pathways were ranked in order of decreasing -log[*p* values]. Regulatory pathways for energy metabolism and cell division progression were among the most significant ones.

**Figure 8:**
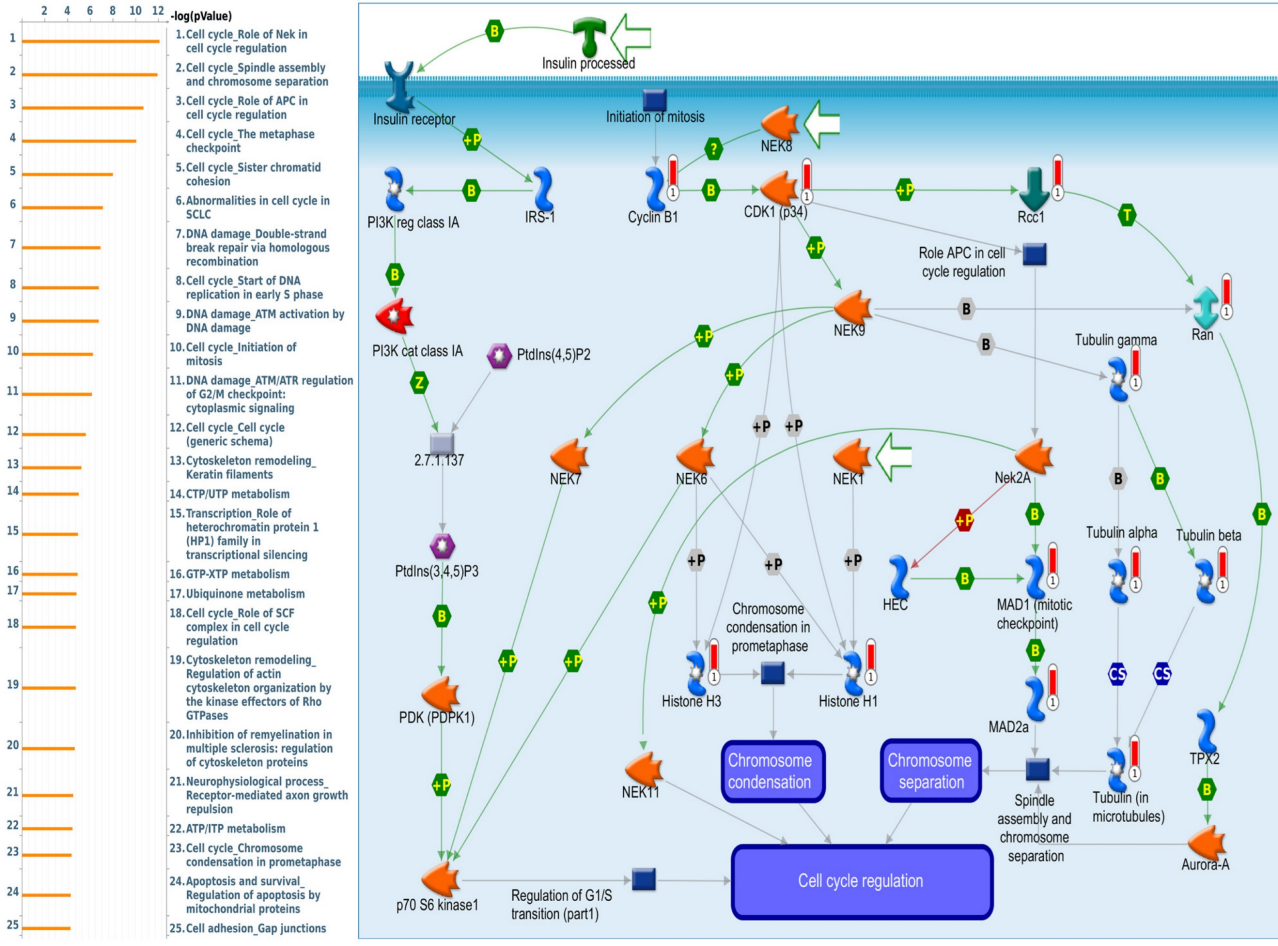
** Significantly associated signaling pathways regulated by top genes coexpressed with *PSMG3* (proteasome assembly chaperone 3), as predicted by MetaCore.** The top 10% coexpressed genes with *PSMG3* shared from TCGA databases were selected for building a gene expression network using "biological processes" of MetaCore. The involved pathways were ranked in order of decreasing -log[*p* values]. Regulatory pathways for cell division progression were among the most significant ones.

**Figure 9:**
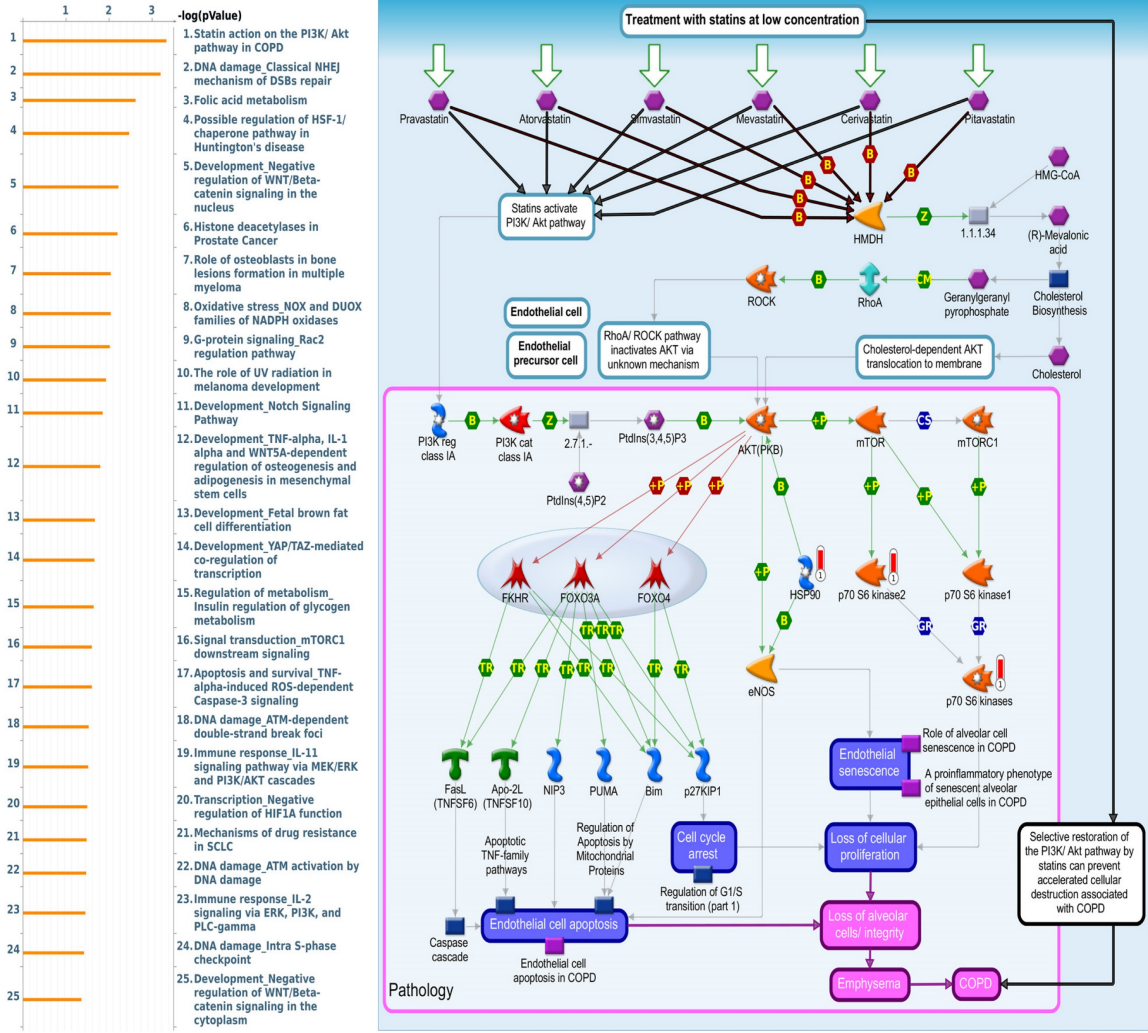
** Significantly associated signaling pathways regulated by top genes coexpressed with *PSMG4* (proteasome assembly chaperone 4), as predicted by MetaCore.** The top 10% coexpressed genes with *PSMG4* shared from TCGA databases were selected for gene expression network predictions using "biological processes" of MetaCore. The involved pathways were ranked in order of decreasing -log[*p* values]. “Statin action on the PI3K Akt pathway in COPD”, “DNA damage_Classical NHE mechanism of DSBs repair”, and “Folic acid metabolism” were among the most significant ones.

Since there is still limited potential information to refine the full picture of regulated pathways available to *PSMG* family genes, the GeneGo MetaCore platform was conducted to the aforementioned coexpression patterns of *PSMG* family genes. We obtained *PSMG1* co-expression profiles in LUAD from both the LUAD TCGA 51 and CPTAC datasets 52. As a result, annotations of almost all biological processes obtained from GeneGo Metacore showed that genes coexpressed with *PSMG1* regulated cell cycle-related pathways such as “Cell cycle_Role of APC in cell cycle regulation”, “Cell cycle_The metaphase checkpoint”, “Ubiquinone metabolism”, “Cell cycle_Spindle assembly and chromosome separation”, and “Cell cycle_Role of Nek in cell cycle regulation” (Figure 6; Supplementary Table S1). *PSMG2* was involved in cancer metabolism-related pathways, including “GTP-XTP metabolism”, “ATP/ITP metabolism”, “Ubiquinone metabolism”, “Cell cycle_Role of APC in cell cycle regulation”, and “CTP/UTP metabolism” (Figure 7; Supplementary Table S2). *PSMG3* was involved in pathways related to “Cell cycle_Role of Nek in cell cycle regulation”, “Cell cycle_Spindle assembly and chromosome separation”, “Cell cycle_Role of APC in cell cycle regulation”, “Cell cycle_The metaphase checkpoint”, and “Cell cycle_Sister chromatid cohesion” (Figure 8; Supplementary Table S3). *PSMG4* was involved in pathways related to “Statin action on the PI3K/Akt pathway in COPD”, “DNA damage_Classical NHEJ mechanism of DSBs repair”, “Folic acid metabolism”, “Possible regulation of HSF-1/chaperone pathway in Huntington's disease”, and “Development_Negative regulation of WNT/Beta-catenin signaling in the nucleus” (Figure 9; Supplementary Table S4).

### PSMG4 mRNA and protein in LUAD cell lines

**Figure 10:**
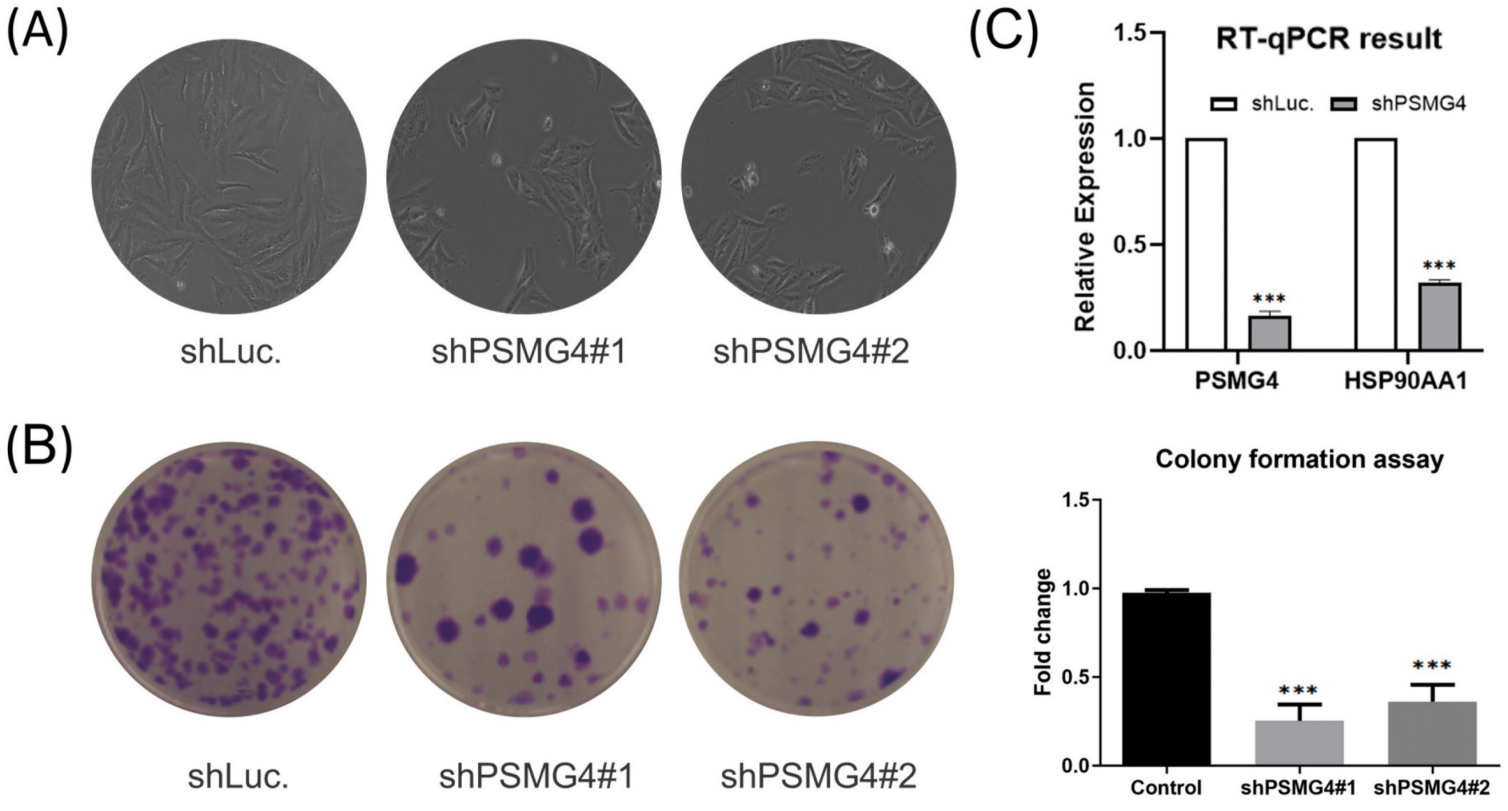
** PSMG4 (proteasome assembly chaperone 4) expression in lung adenocarcinoma (LUAD) cell lines. (A)** Bright-field microscopic images of A549-shLuc as the control, along with two A549-shPSMG4 counterparts, using two-dimensional cell culture modes. Spindle-like morphology of the A549-shLuc control became epithelial-like after PSMG4 silencing. **(B)** RT-qPCR showing decreased mRNA expression levels of HSP90AA1--an important regulator of autophagy involved in downstream signaling pathways regulated by *PSMG4*. Data were normalized against 18S rRNA as an internal control using the 2^-ΔΔCt^ method. Statistical significance was determined by Student's *t*-test and error bars represent the SD (* *p* < 0.05, ** *p* < 0.01) of duplicate determinations. **(C)** Colony-formation assay. Representative micrographs (left) and quantification (right) of crystal violet-stained clones confirmed the influence of *PSMG4* on attenuating A549 cell proliferation. The long-term proliferation of two A549 shPSMG4-knockdown cell lines markedly declined compared to A549-shLuc as the control.

The above bioinformatics analysis indicated that *PSMG4* expression was higher in tumor samples compared to normal samples, and further promoted development advance in the tumor stage via the heat shock protein 90 (HSP90)/phosphatidylinositol 3-kinase (PI3K)/AKT/Wnt signaling pathway. Therefore, among *PSMG* family members, we chose *PSMG4* for further study. Intriguingly, in the absence of *PSMG4* in A549 cells, cells changed to a more-cuboidal (epithelial-like) morphology compared to shLuciferase control cells (Figure 10A). Lipofectamine transfection of PSMG4-shRNA was performed to inhibit *PSMG4* expression in LUAD cells. The suppressive efficacy was confirmed by a qPCR (Figure 10B). Meanwhile, expression levels of an *HSP90*-related marker (*HSP90AA1*) decreased after *PSMG4* expression was downregulated (Figure 10B). We investigated long-term cell proliferation by a colony-formation assay, and found that the cellular proliferation in *PSMG4*-silenced LUAD cells had decreased (Figure 10C).

## Discussion

Recent epidemiologic studies indicated that lung cancer remains at the top of fatal malignancies, despite remarkable improvements that have been made in medical and surgical approaches. As a matter of fact, shortages of highly sensitive screening tests, delays in early screening, and high probabilities of drug and chemoresistance have resulted in increased risks of metastasis and relapse, as well as meager survival rates for lung cancer patients. Therefore, identifying specific and highly sensitive biomarkers for lung cancer is urgent, with the goal of formulating effective treatments known as personalized medicine 53.

To date, the rapid growth of microarray and high-throughput sequencing techniques has generated enormous amounts of data available in either web-based platforms or comprehensive tools that allow us to properly monitor tumor progression at various levels, including genomics, epigenomics, transcriptomics, proteomics, and metabolomics 54, 55. The underlying pathogenesis of tumors in general and lung cancer, in particular, has thus been reported to be dominated by specific somatic genomic alterations. Notably, *KRAS, BRAF, ERBB2, PIK3CA, RET, ROS1, ALK*, and *NRTK1/2/3* have been proposed as prognostic markers, making substantial contributions to genotype-directed therapies for advanced lung cancer. By applying for advances in high-throughput screening on cancer transcriptomic profiling, alterations in transcriptome patterns of gene families encoded for human proteasomes were discovered to be significantly associated with several types of malignancies, among which *PSMG* gene expressions were found to be involved not only in multi-stage tumor progression but also in other tumor-related aspects. However, since previous research has yet to elaborate on the roles of *PSMG* family genes in LUAD, this study can serve as the first and foremost work that specifically examined the roles of *PSMG* individuals in LUAD.

PSMG1 and PSMG2, previously referred to as PAC1 and PAC2, are two evolutionarily conserved, ubiquitously expressed chaperone proteins promoting proper assembly of the α-ring of the 20S CP human proteasome 56. PSMG1 assembles in a complex with PSMG2 since intact PSMG1-PSMG2 heterodimers help to stabilize the assembly of the heteroheptameric α-ring and prevent accumulation of non-productive α-ring dimers 57. The previous literature confirmed that PSMG1 plays significant roles in increasing susceptibility to inflammatory bowel diseases, a key factor leading to a higher risk of colorectal cancer, which can be explained by the close location of rs2836878 to PSMG1 supporting their function in the ubiquitin-proteasome system 58-61. In addition, targeting PSMG1 caused by miR-484 inhibition led to reductions in cell migration and invasion in prostate cancer 62. In this study, analytical results confirmed elevated expression of the PSMG1 transcriptome in LUAD tissues compared to normal ones. Furthermore, incremental expression of the *PSMG1* gene was recorded in advanced stages of LUAD, in both individuals and nodal statuses, and especially in patients bearing the *TP53* mutation, suggesting that a regulatory axis exists between *PSMG1* and other cellular factors that drive the progression of LUAD. Subsequent findings also designated the “Cell cycle_Role of APC in cell cycle regulation” as a dominant pathway presented in the coexpression gene network of *PSMG1*. The findings mentioned above are consistent with previous knowledge of *PSMG1*'s roles; however, survival analysis showed no signs of a relationship between elevated levels of the *PSMG1* transcriptome and FP rates of patients with LUAD; and a limited number of IHC staining samples came back positive for *PSMG1*, demonstrating the minor presence of this gene in biopsies of LUAD patients.

The distinct roles in human diseases of *PSMG2*, on the other hand, were largely unknown, apart from the monogenic inheritance of CANDLE (chronic atypical neutrophilic dermatosis with lipodystrophy and elevated temperature) syndrome that was previously reported to occur of the *PSMG2* mutations 63. As specified by the MetaCore enrichment pathway analysis, GTP, XTP, ATP, ITP, and ubiquinone-related metabolism signaling are among *PSMG2* coexpressed pathways with the most significant *p* values; however, there were no noticeable changes recorded in gene expression levels either in later stages, nodal statuses, or in the *TP53*-mutated population. Similarly, a subsequent immune infiltration analysis also did not signify substantial correlations between expression patterns of specific immune cells and *PSMG2* transcriptomics. Such observations suggest that *PSMG2* seems to only play a minor role in LUAD progression.

*PSMG3* and *PSMG4*, alternatively known as *PAC3* and *PAC4*, are mainly regulate the recruitment of the β-ring 64. Given that malfunctions of these assembly chaperones cause the accumulation of imperfectly assembled or misassembled complexes of proteasomal subunits, knockdown experiments involving *PSMG3* and *PSMG4* resulted in the accumulation of abnormal α-subunit oligomers 65. Elevated *PSMG3* levels found in the plasma of glioblastoma multiforme (GBM) patients help distinguish those with sarcoidosis and healthy patients 50. In addition, a significant number of lncRNA-PSMG3 and miRNA-PSMG3 axis regulatory mechanisms were reported to be associated with malignant diseases, including lung cancer 66-69. On the contrary, limited literature has described the relationship between *PSMG4* and human diseases, especially tumors. In our study, expressions of *PSMG3* and *PSMG4* were both found to progressively remain elevated toward increasing advanced stages of individuals, nodal statuses, and also in LUAD patients with the *TP53* mutation. The survival analysis indicated that upregulation of *PSGM3* and *PSMG4* resulted in poor prognoses, in terms of OS in general and both FP and PPS in particular.

Subsequent analyses of *PSMG4*, in particular, showed strong consistency with earlier studies. First, abundant expression of the PSMG4 protein was found in LUAD tissues through IHC staining, compared to the normal adjacent lung tissues. Second, regulatory pathways predicted based on the top genes coexpressed with *PSMG4* suggested that the top three most significant pathways included “Statin action on the PI3K Akt pathway in COPD”, “DNA damage_Classical NHE mechanism of DSBs repair”, and “Folic acid metabolism”, which may partly explain why *PSMG4* plays a key role in LUAD. To be more specific, DNA damage, particularly DNA double-strand breaks (DSBs), is highly deleterious since a single unrepaired DSB is sufficient to trigger cellular senescence or apoptosis, and DSB-repair and associated sub-pathways play key roles in suppressing tumorigenesis. However, the relationship between folic acid intake and lung cancer risk remains controversial due to biased evidence among genders and ages 70-72. Most remarkable of all, there is ample evidence that dysregulation of cholesterol homeostasis, accumulation of cholesteryl ester-rich lipid droplets in lung tissues, and NSCLC are intimately linked. Interestingly, statin treatment was observed to show antitumorigenic activities against lung cancer by suppressing AKT and the Braf/mitogen-activated protein kinase kinase (MEK)/extracellular signal-regulated kinase 1/2 (ERK1/2) pathways 73-75. Moreover, numerous experiments on the NSCLC-derived A459 cell line revealed that p-Akt inhibition helped increase apoptosis and reduce radio-resistance 75-77, and bioinformatics data also presented DNA methylation expression levels of *PSMG4* in lung cancer (Supplementary Figure S2). Third, our wet lab validation indicated that silencing of the *PSMG4* gene not only helped attenuate proliferation of tumor cells, but also significantly reduced the mRNA level of heat shock protein 90 (*HSP90AA1*) - a cytosolic protein that belongs to the *HSP90* family, which was in agreement with the downstream signaling pathway predicted by MetaCore (Figure 9).

As a chaperone protein well-known for its role in stabilizing various growth factor receptors (such as tumor necrosis factor receptors (TNFRs) and NF-κB) and various signaling molecules found overexpressed in tumor cells (including PI3K and AKT proteins), inhibition of *HSP90* was reported to downregulate the PI3K/AKT pathway, thus resulting in apoptosis of tumor cells via downregulation of the antiapoptotic protein, BCL-w 78-81. In addition, *HSP90* also participates in a significant number of key processes in *de novo* angiogenesis which is necessary for tumor growth under hypoxia, and promotes tumor metastasis 82-84. Most interestingly, the relationship of the *HSP90* protein and intrinsic resistance to immunotherapies by tumors has been of interest since previous studies indicated that specific inhibition of *HSP90* helps drive potent T-cell responses in the TME, specifically reducing surface expressions of several immune checkpoint proteins (such as programmed death ligand 1 (PD-L1) and PD-L2), reducing the number of regulatory T (Treg) cells, and stimulating “killer” T cells (CD8^+^ cytotoxic T-lymphocytes (CTLs)) and “helper” T cells (CD4^+^) 85-87, thereby triggering an immunosuppressive TME and relieving tumor immune escape. Similarly, such immune infiltration patterns of *PSMG4* were recorded in LUAD patients via a TIMER analysis, which neatly supports the strong biological associations between *PSMG4* and *HSP90AA1* in the context of lung cancer.

Taken together, our study proposed that elevated expression of *PSMG4* leads to dismal outcomes for LUAD patients, whereas inhibition of *PSMG4* resulted in a reduced cell proliferation rate, along with a marked decline in the expression level of the *HSP90AA1* protein. Additionally, similar immune infiltration patterns between *PSMG4* and *HSP90AA1* also suggested that *PSMG4* may play a certain role in the immune-suppressive TME which is common among lung cancer patients. *PSMG4* can thus possibly serve as an independent risk factor and immuno-prognostic factor for LUAD patients. However, detailed signaling pathways are mandated to figure out the immune crosstalk, hence allowing better guidance for clinical immunotherapy and providing accurate personalized treatment plans for LUAD patients.

## Conclusions

To summarize, our study proposed *PSMG4* as a potential prognostic marker and therapeutic target of LUAD. Knockdown of *PSMG4* in a LUAD cell line reduced cell proliferation and influenced expressions of downstream molecules, including *HSP90*-related genes. The current study revealed that *PSMG4*-related signaling may potentially be targeted for the treatment of LUAD. Further in-depth experiments are mandated to extensively explore *PSMG4*'s key roles in the underlying pathogenesis and immune crosstalk in LUAD progression and metastatic spread that participate in cancer development.

## Supplementary Material

Supplementary figures and tables.
