# Supplementary material for: Prognostic and Immune Infiltration Value of Proteasome Assembly Chaperone (PSMG) Family Genes in Lung Adenocarcinoma
Source: Int J Med Sci. 2023 Jan 1;20(1):87–101. doi: 10.7150/ijms.78590 (PMC9812804; doi:10.7150/ijms.78590)
Supplement: Supplementary file 1 — Supplementary figures and tables. [file ijmsv20p0087s1.pdf]

**Supplementary data:**

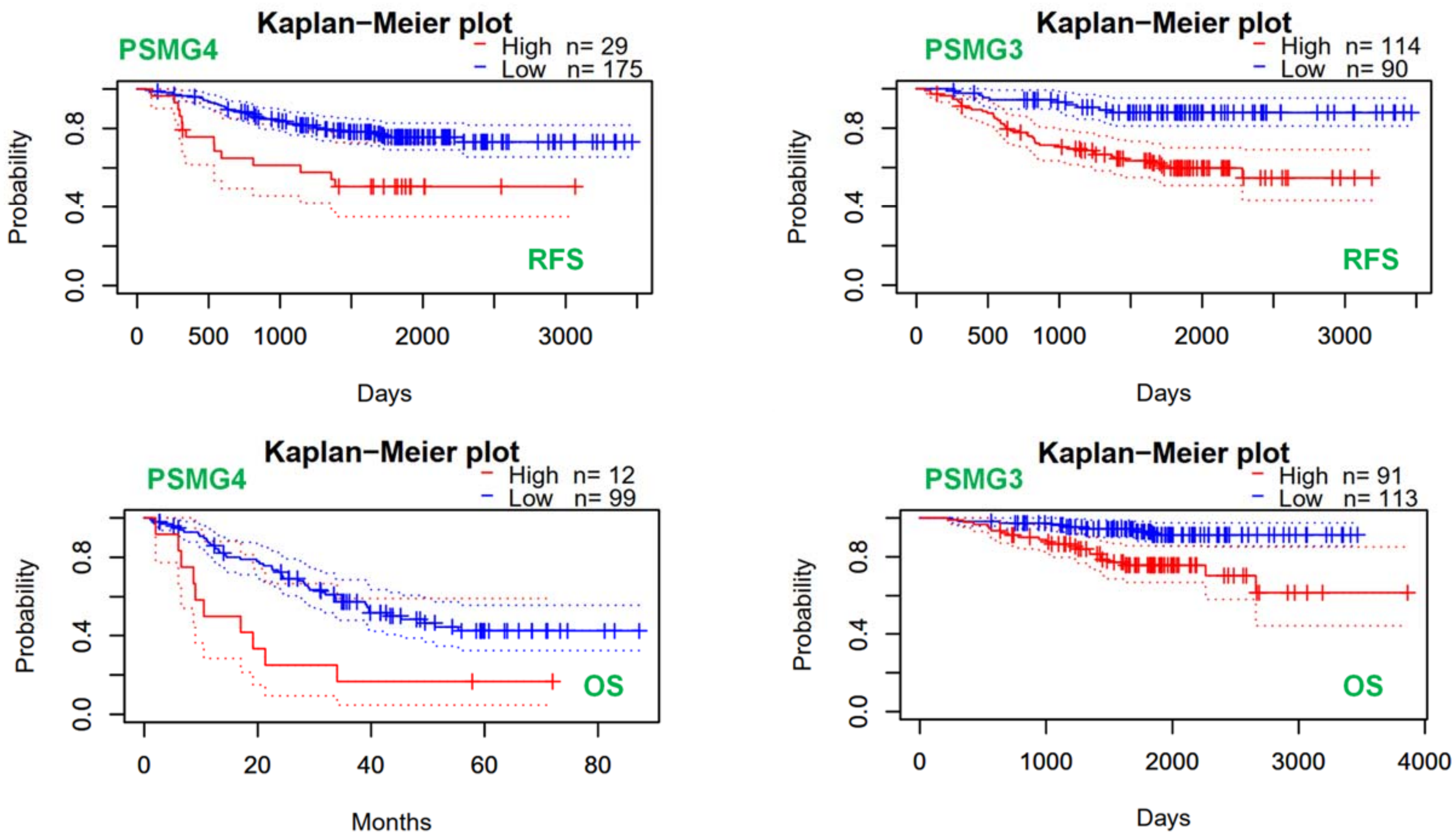


Figure S1: **Predicted survival analyses according to expression level of PSMG3 and PSMG4 in LUAD patient via prognoscan database.**

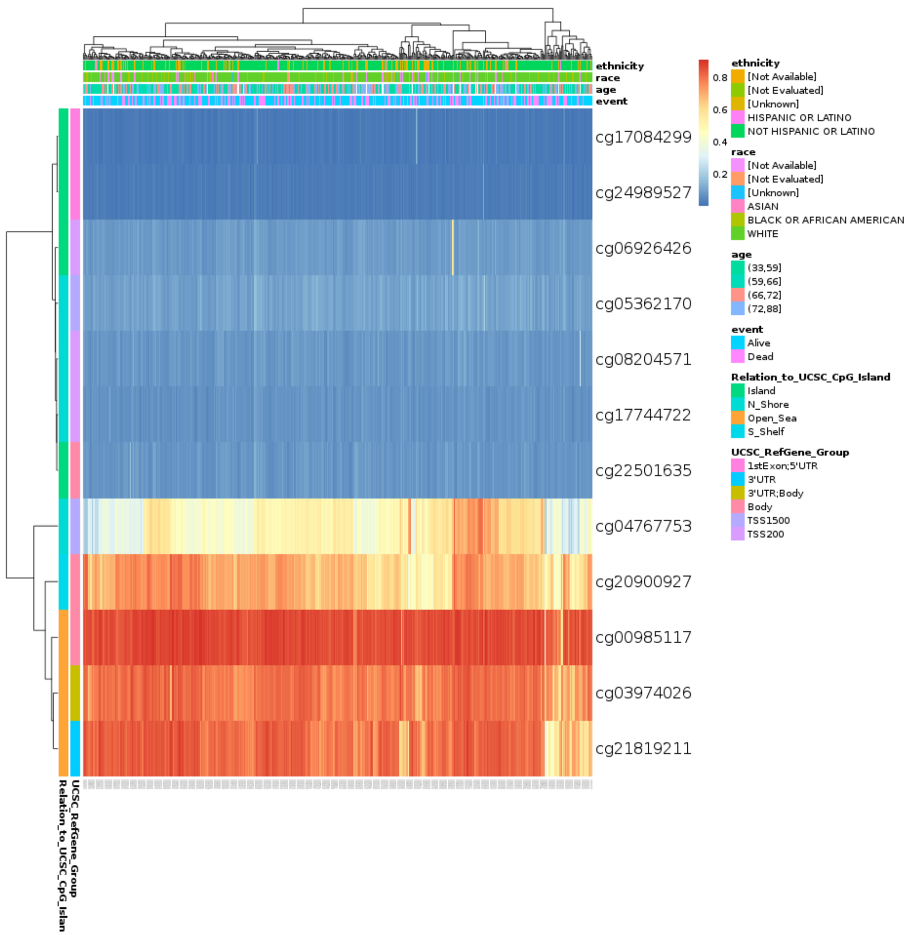


**Figure S2: Heatmap of DNA methylation expression levels of the PSMG4 in lung cancer by MethSurv platform.**

**Table S1: Pathway analysis of genes co-expressed with PSMG1 from public LUAD databases using the MetaCore database (with *p*<0.01 set as the cutoff value)**

| # | Maps | pValue | Network Objects from Active Data |
|---|---|---|---|
| 1 | Cell cycle_Role of APC in cell cycle regulation | 2.16E-21 | Nek2A, BUB1, MAD2b, BUB3, Tome-1, Geminin, Emi1, Cyclin A, Aurora-A, TCP1, Aurora-B, Kid, CDC20, Cyclin B, MAD2a, Securin, CDK1 (p34), CKS1 |
| 2 | Cell cycle_The metaphase check-point | 1.52E-18 | Nek2A, BUB1, MAD2b, SPBC25, CENP-A, BUB3, DSN1, Aurora-A, Aurora-B, HP1 gamma, HEC, CDCA1, CDC20, HZwint-1, MAD2a, Survivin, CENP-H |
| 3 | Ubiquinone metabolism | 2.03E-18 | NDUFS5, NDUFAB1, NDUFA1, NDUFB9, NDUFB4, NDUFA8, NDUFA9, NDUFB6, NDUFB3, COQ3, NDUFB2, NDUFS8, NDUFB1, NDUFS6, coenzyme Q2 homolog, prenyltransferase (yeast), NDUFV1, NDUFS3, NDUFB10, NDUFC2, NDUFC1, DAP13, NDUFV3 |
| 4 | Cell cycle_Spindle assembly and chromosome separation | 1.14E-14 | Nek2A, CSE1L, RCC1, Aurora-A, Aurora-B, HEC, Kid, CDC20, Tubulin alpha, Cyclin B, MAD2a, Securin, Ran, CDK1 (p34) |
| 5 | Cell cycle_Role of Nek in cell cycle regulation | 5.54E-12 | Nek2A, Tubulin gamma, Cyclin B1, RCC1, Aurora-A, HEC, Tubulin alpha, MAD2a, Ran, Histone H3, Histone H1, CDK1 (p34) |
| 6 | DNA damage_ATM/ATR regulation of G2/M checkpoint: cytoplasmic signaling | 1.85E-10 | CDC25C, UBE2C, Cyclin B1, JAB1, Chk2, Chk1, Aurora-A, PP1-cat, Aurora-B, PP2A catalytic, Histone H3, CDK1 (p34), 14-3-3 |
| 7 | Cell cycle_Chromosome condensa-tion in prometaphase | 6.46E-10 | CAP-G, Cyclin A, CAP-G/G2, Aurora-A, Aurora-B, Cyclin B, Histone H3, Histone H1, CDK1 (p34) |
| 8 | Abnormalities in cell cycle in SCLC | 8.98E-10 | CDK4, PCNA, Cyclin B1, Cyclin A, Cyclin E, Aurora-B, Histone H3, CDK1 (p34), Cyclin E2, CKS1 |
| 9 | Cell cycle_Start of DNA replication in early S phase | 2.71E-09 | RPA3, Geminin, Cyclin E, ORC6L, ORC5L, ASK (Dbf4), PP2A catalytic, CDC7, Histone H1, CDC45L |
| 10 | GTP-XTP metabolism | 3.57E-08 | POLR2J, NDPK A, POLR2I, RRP41, RPB6, RRP46, HPRT, RPB7.0, POLR3K, POLR3F, RPB8, POLR2G, RPA16, RPA39 |
| 11 | ATP/ITP metabolism | 5.12E-08 | POLR2J, NDPK A, POLR2I, RRP41, RPB6, RRP46, HPRT, RPB7.0, POLR3K, APRT, ADSL, POLR3F, RPB8, POLR2G, RPA16, RPA39 |
| 12 | DNA damage_ATM/ATR regulation of G2/M checkpoint: nuclear signal-ing | 1.01E-07 | CDC25C, Cyclin B1, Histone H2AX, Chk2, Cyclin A, Chk1, Cyclin B, Cyclin B2, TTK, CDK1 (p34) |
| 13 | DNA damage_Double-strand break repair via homologous recombina-tion | 1.18E-07 | MAD2b, DSS1, Histone H2AX, PPP4C, Rad51, RAD54B, SWI5, PIR51, Histone H2A, E2N(UBC13), AUNIP, RAD51C, CDK1 (p34) |
| 14 | Role of XBP1 protein in multiple myeloma | 2.93E-07 | SERP1, PSMA5, DNAJB11, PSMA6, PSMA7, Proteasome (20S core), ERP5 |
| 15 | CTP/UTP metabolism | 3.3E-07 | POLR2J, CTP synthase, NDPK A, POLR2I, RRP41, RPB6, RRP46, RPB7.0, POLR3K, POLR3F, RPB8, POLR2G, RPA16, RPA39 |

| | | | |
|---|---|---|---|
| **16** | Cell cycle_Cell cycle (generic schema) | 4.3E-07 | CDC25C, CDK4, Cyclin A, Cyclin E, Cyclin B, E2F6, CDK1 (p34) |
| **17** | Immune response_Antigen presentation by MHC class I, classical pathway | 6.23E-07 | PSMB1, PSMB5, PSME2, BCAP31, TCP1, PA28 (11S regulator), Impas 1, PSMB2, PSME1, Proteasome (20S core) |
| **18** | Cell cycle_Sister chromatid cohesion | 8.69E-07 | PCNA, Cyclin B, DCC1, Securin, Histone H3, Histone H1, CDK1 (p34) |
| **19** | Putative pathways of activation of monoclonal protein secretion in multiple myeloma | 1.63E-06 | SSR-delta, SERP1, DAD-1, DNAJB11, Cyclophilin B, ARMET, ERP5 |
| **20** | DNA damage_Intra S-phase checkpoint | 2.23E-06 | PCNA, Histone H2AX, Chk2, Cyclin A, Chk1, PP1-cat, PP1-cat alpha, ASK (Dbf4), CDC7, Histone H3, CDC45L |
| **21** | Cell cycle_Role of SCF complex in cell cycle regulation | 4.87E-06 | CDK4, Emi1, Cyclin E, Chk1, NEDD8, CDK1 (p34), CKS1 |
| **22** | DNA damage_ATM/ATR regulation of G1/S checkpoint | 9.78E-06 | CDK4, PCNA, Histone H2AX, Chk2, Cyclin A, Cyclin E, Chk1, PP2A catalytic |
| **23** | Cell cycle_Nucleocytoplasmic transport of CDK/Cyclins | 1.43E-05 | CDK4, Cyclin B1, Cyclin A, Cyclin E, CDK1 (p34) |
| **24** | CREB1-dependent transcription deregulation in Huntington's Disease | 3.14E-05 | Cytochrome c, COX VIIc, TFAM, NDUFS3, COX VIa-1, SOD1 |
| **25** | Cell cycle_Initiation of mitosis | 3.14E-05 | CDC25C, Cyclin B1, Cyclin B2, Histone H3, Histone H1, CDK1 (p34) |

**Table S2: Pathway analysis of genes co-expressed with PSMG2 from public LUAD databases using the MetaCore database (with *p*<0.01 set as the cutoff value)**

| **#** | **Maps** | **pValue** | **Network Objects from Active Data** |
|---|---|---|---|
| **1** | GTP-XTP metabolism | 1.72E-09 | RRP4, PM/SCL-75, NDPK A, NDPK complex, RRP43, RPB7.0, RRP40, NDPK B, RPB8, CSL4, POLR2G, PNPH, RPA16, RPA39 |
| **2** | ATP/ITP metabolism | 1.40E-08 | RRP4, PM/SCL-75, NDPK A, NDPK complex, RRP43, RPB7.0, RRP40, ADSL, NDPK B, RPB8, CSL4, POLR2G, PNPH, RPA16, RPA39 |
| **3** | Ubiquinone metabolism | 1.58E-08 | NDUFAB1, NDUFA1, NDUFB4, NDUFA8, NDUFA9, NDUFB6, NDUFB3, NDUFB5, NDUFV2, NDUFA5, NDUFS3, DAP13 |
| **4** | Cell cycle_Role of APC in cell cycle regulation | 1.22E-07 | BUB3, Geminin, Cyclin A, Cyclin B, MAD2a, Securin, CDK1 (p34), CKS1 |
| **5** | CTP/UTP metabolism | 1.38E-07 | RRP4, PM/SCL-75, NDPK A, NDPK complex, RRP43, RPB7.0, RRP40, NDPK B, RPB8, CSL4, POLR2G, RPA16, RPA39 |

| | | | |
|---|---|---|---|
| **6** | Cell cycle_Spindle assembly and chromosome separation | 1.59E-07 | Ubiquitin, HEC, Tubulin alpha, Cyclin B, MAD2a, Securin, Ran, CDK1 (p34) |
| **7** | DNA damage_ATM/ATR regulation of G2/M checkpoint: nuclear signaling | 2.02E-06 | CDC25C, RBBP8 (CtIP), Cyclin B1, Ku70, Cyclin A, Cyclin B, Cyclin B2, CDK1 (p34) |
| **8** | Cell cycle_The metaphase checkpoint | 4.78E-06 | BUB3, HP1 gamma, HEC, CDCA1, MAD2a, Survivin, CENP-H |
| **9** | Apoptosis and survival_Granzyme A signaling | 1.19E-05 | NDPK A, HSP70, HMGB2, hnRNP C, Ku70, APEX, NDUFS3 |
| **10** | LRRK2 in neuronal apoptosis in Parkinson's disease | 1.32E-05 | Cytochrome c, PRDX3, Thioredoxin, ANT, Caspase-3 |
| **11** | Cell cycle_Role of Nek in cell cycle regulation | 2.91E-05 | Cyclin B1, HEC, Tubulin alpha, MAD2a, Ran, CDK1 (p34) |
| **12** | DNA damage_Double-strand break repair via homologous recombination | 3.38E-05 | NARF, RBBP8 (CtIP), DSS1, Rad51, SWI5, Histone H2A, E2N(UBC13), SFR1, CDK1 (p34) |
| **13** | DNA damage_ATM/ATR regulation of G2/M checkpoint: cytoplasmic signaling | 5.18E-05 | CDC25C, MLCP (cat), Cyclin B1, PP1-cat, PP2A catalytic, CDK1 (p34), IPP-2 |
| **14** | Proteolysis_Putative ubiquitin pathway | 6.55E-05 | Ubiquitin, HSP70, UBCH6, E2N(UBC13), UBCH7 |
| **15** | Cell cycle_Sister chromatid cohesion | 6.55E-05 | PCNA, Cyclin B, RFC3, Securin, CDK1 (p34) |
| **16** | IL-6 signaling in colorectal cancer | 6.89E-05 | Cyclin B1, HSP70, Ku70, Cyclin B, Survivin, CDK1 (p34) |
| **17** | Immune response_Antigen presentation by MHC class I, classical pathway | 7.54E-05 | PSMB1, PSMB5, PSME2, HSP70, PA28 (11S regulator), PSME1, PSMB8(LMP7) |
| **18** | Transcription_Role of heterochromatin protein 1 (HP1) family in transcriptional silencing | 1.08E-04 | SUMO-1, Cyclin A2, HP1 gamma, MBD2, HP1, CDK1 (p34) |
| **19** | Cell cycle_Initiation of mitosis | 1.22E-04 | CDC25C, Cyclin B1, Cyclin B2, CDK1 (p34), CDK7 |
| **20** | Translation_(L)-selenoaminoacids incorporation in proteins during translation | 1.25E-04 | SEP15, Selenoprotein K, RPL30, SelT, SEPHS1, SELS |
| **21** | Inhibition of remyelination in multiple sclerosis: regulation of cytoskeleton proteins | 1.87E-04 | MLCP (cat), CDK5, Tubulin alpha, MRLC, Stathmin, Cofilin |

| | | | |
|---|---|---|---|
| **22** | Abnormalities in cell cycle in SCLC | 2.10E-04 | PCNA, Cyclin B1, Cyclin A, CDK1 (p34), CKS1 |
| **23** | dGTP metabolism | 2.25E-04 | NDPK A, POLE3 (YBL1), NDPK complex, DGUOK, NDPK B, PNPH, POLE4 |
| **24** | Immune response_BAFF-induced non-canonical NF-kB signaling | 2.48E-04 | Ubiquitin, SUMO-1, UBE1C, E2N(UBC13), NEDD8 |
| **25** | dATP/dITP metabolism | 4.62E-04 | NDPK A, POLE3 (YBL1), NDPK complex, DGUOK, ADSL, NDPK B, PNPH, POLE4 |

**Table S3: Pathway analysis of genes co-expressed with PSMG3 from public LUAD databases using the MetaCore database (with *p*<0.01 set as the cutoff value)**

| # | Maps | pValue | Network Objects from Active Data |
|---|---|---|---|
| **1** | Cell cycle_Role of Nek in cell cy-cle regulation | 8.06E-13 | Tubulin beta, Tubulin gamma, Cyclin B1, RCC1, MAD1 (mitotic checkpoint), Tubulin alpha, MAD2a, Ran, Histone H1, Histone H3, CDK1 (p34), Tubulin (in microtubules) |
| **2** | Cell cycle_Spindle assembly and chromosome separation | 1.24E-12 | RCC1, MAD1 (mitotic checkpoint), Aurora-B, Kid, CDC20, Tubulin alpha, Cyclin B, MAD2a, Securin, Ran, CDK1 (p34), Tubulin (in microtubules) |
| **3** | Cell cycle_Role of APC in cell cy-cle regulation | 2.27E-11 | MAD2b, Tome-1, Geminin, Aurora-B, Kid, CDC20, Cyclin B, MAD2a, Securin, CDK1 (p34), CKS1 |
| **4** | Cell cycle_The metaphase checkpoint | 9.8E-11 | MAD2b, SPBC25, CENP-A, MAD1 (mitotic checkpoint), Aurora-B, HP1 gamma, CDCA1, CDC20, MAD2a, Survivin, CENP-H |
| **5** | Cell cycle_Sister chromatid cohe-sion | 1.19E-08 | PCNA, CHTF18, Cyclin B, DCC1, Securin, Histone H1, Histone H3, CDK1 (p34) |
| **6** | Abnormalities in cell cycle in SCLC | 9.33E-08 | CDK4, PCNA, Cyclin B1, Cyclin E, Aurora-B, Histone H3, CDK1 (p34), CKS1 |
| **7** | DNA damage_Double-strand break repair via homologous re-combination | 1.54E-07 | MAD2b, RecQL4, WDR79, DSS1, Histone H2AX, PPP4C, Rad51, SWI5, Histone H2A, AUNIP, His-tone H4, CDK1 (p34) |
| **8** | Cell cycle_Start of DNA replica-tion in early S phase | 2.16E-07 | RPA3, Geminin, Cyclin E, ORC6L, ORC5L, ASK (Dbf4), Histone H1, CDC45L |
| **9** | DNA damage_ATM activation by DNA damage | 2.19E-07 | HSP90, OBFC2B, RecQL4, HMG14, Histone H2AX, PP2A regulatory, CDK5, Suv39H1, Histone H4, BRAT1, Histone H3 |
| **10** | Cell cycle_Initiation of mitosis | 7.34E-07 | CDC25C, Cyclin B1, Cyclin B2, Kinase MYT1, Histone H1, Histone H3, CDK1 (p34) |
| **11** | DNA damage_ATM/ATR regula-tion of G2/M checkpoint: cyto-plasmic signaling | 9.08E-07 | CDC25C, UBE2C, Cyclin B1, JAB1, PP2A regulatory, PP1-cat, Aurora-B, Histone H3, CDK1 (p34) |
| **12** | Cell cycle_Cell cycle (generic schema) | 3.21E-06 | CDC25C, CDK4, Cyclin E, Cyclin B, E2F4, CDK1 (p34) |

| | | | |
|---|---|---|---|
| **13** | Cytoskeleton remodeling_Keratin filaments | 7.79E-06 | Tubulin beta, Tubulin gamma 1, Keratin 18, Actin cytoskeletal, Tubulin alpha, CDK1 (p34), Tubulin (in microtubules) |
| **14** | CTP/UTP metabolism | 1.37E-05 | POLR2J, NDPK A, POLR2I, RRP41, RPB6, RPB7.0, RPB8, UDP, RPA16, RPA39, NDPK D (mitochondrial) |
| **15** | Transcription_Role of heterochromatin protein 1 (HP1) family in transcriptional silencing | 1.62E-05 | Cyclin E, HP1 gamma, HP1, Suv39H1, Histone H4, Histone H3, CDK1 (p34) |
| **16** | GTP-XTP metabolism | 1.71E-05 | POLR2J, NDPK A, POLR2I, RRP41, RPB6, RPB7.0, RPB8, RPA16, RPA39, NDPK D (mitochondrial) |
| **17** | Ubiquinone metabolism | 2.17E-05 | NDUFAB1, NDUFA1, NDUFA4, COQ3, NDUFS8, NDUFS6, coenzyme Q2 homolog, prenyltransferase (yeast), NDUFV1, DAP13 |
| **18** | Cell cycle_Role of SCF complex in cell cycle regulation | 2.44E-05 | CDK4, Cyclin E, NEDD8, CDK1 (p34), CDC34, CKS1 |
| **19** | Cytoskeleton remodeling_Regulation of actin cytoskeleton organization by the kinase effectors of Rho GTPases | 2.47E-05 | ARPC1B, Actin cytoskeletal, Rac3, Cofilin, non-muscle, F-Actin cytoskeleton, Cofilin, Rac1-related, Rac1 |
| **20** | Inhibition of remyelination in multiple sclerosis: regulation of cytoskeleton proteins | 3.1E-05 | Tubulin beta, Actin cytoskeletal, CDK5, Tubulin alpha, Cofilin, Rac1, Tubulin (in microtubules) |
| **21** | Neurophysiological process_Receptor-mediated axon growth repulsion | 4.18E-05 | Ephrin-A, Actin cytoskeletal, CDK5, F-Actin cytoskeleton, Cofilin, Rac1, Tubulin (in microtubules) |
| **22** | ATP/ITP metabolism | 5.08E-05 | POLR2J, NDPK A, POLR2I, RRP41, RPB6, RPB7.0, ADSL, RPB8, RPA16, RPA39, NDPK D (mitochondrial) |
| **23** | Cell cycle_Chromosome condensation in prometaphase | 5.83E-05 | Aurora-B, Cyclin B, Histone H1, Histone H3, CDK1 (p34) |
| **24** | Apoptosis and survival_Regulation of apoptosis by mitochondrial proteins | 7.1E-05 | Cytochrome c, GC1QBP, MTCH2, HtrA2, TIMM8A, Metaxin 1, Smac/Diablo, PP1-cat alpha, PP2C, Cofilin |
| **25** | Cell adhesion_Gap junctions | 7.42E-05 | Tubulin beta, Actin cytoskeletal, Actin, Tubulin alpha, Tubulin (in microtubules) |

**Table S4: Pathway analysis of genes co-expressed with PSMG4 from public LUAD databases using the MetaCore database (with *p*<0.01 set as the cutoff value)**

| # | Maps | pValue | Network Objects from Active Data |
|---|---|---|---|
| 1 | Statin action on the PI3K/ Akt pathway in COPD | 4.67E-04 | HSP90, p70 S6 kinase2, p70 S6 kinases |

| | | | |
|---|---|---|---|
| **2** | DNA damage_Classical NHEJ mechanism of DSBs repair | 6.38E-04 | PNKP, DNA polymerase lambda, PAXX |
| **3** | Folic acid metabolism | 2.50E-03 | FOLC1, FOLC2, SLC19A1 |
| **4** | Possible regulation of HSF-1/ chaperone pathway in Huntington's disease | 3.52E-03 | HSP90, HSF1 |
| **5** | Development_Negative regulation of WNT/Beta-catenin signaling in the nucleus | 6.34E-03 | Nitrilase 1, KDM2, RUVBL2 |
| **6** | Histone deacetylases in Prostate Cancer | 6.66E-03 | HSP90, Sirtuin5 |
| **7** | Role of osteoblasts in bone lesions formation in multiple myeloma | 9.61E-03 | Osteocalcin, TAZ |
| **8** | Oxidative stress_NOX and DUOX families of NADPH oxidases | 9.61E-03 | PDIP38, NOXA1 |
| **9** | G-protein signaling_Rac2 regulation pathway | 1.01E-02 | NOXA1, PREX1 |
| **10** | The role of UV radiation in melanoma development | 1.24E-02 | XRCC3, USF1 |
| **11** | Development_Notch Signaling Pathway | 1.49E-02 | Radical fringe, GCN5 |
| **12** | Development_TNF-alpha, IL-1 alpha and WNT5A-dependent regulation of osteogenesis and adipogenesis in mesenchymal stem cells | 1.69E-02 | Osteocalcin, TAZ |
| **13** | Development_Fetal brown fat cell differentiation | 2.27E-02 | p70 S6 kinase2, USF1 |
| **14** | Development_YAP/TAZ-mediated co-regulation of transcription | 2.35E-02 | Osteocalcin, TAZ |
| **15** | Regulation of metabolism_Insulin regulation of glycogen metabolism | 2.43E-02 | PHK gamma, PHK gamma (liver) |
| **16** | Signal transduction_mTORC1 downstream signaling | 2.67E-02 | p70 S6 kinase2, p70 S6 kinases |
| **17** | Apoptosis and survival_TNF-alpha-induced ROS-dependent Caspase-3 signaling | 2.67E-02 | NOXA1, FLAD1 |
| **18** | DNA damage_ATM-dependent double-strand break foci | 3.19E-02 | KDM2A, GCN5 |
| **19** | Immune response_IL-11 signaling pathway via MEK/ERK and PI3K/AKT cascades | 3.28E-02 | Osteocalcin, p70 S6 kinases |
| **20** | Transcription_Negative regulation of HIF1A function | 3.46E-02 | HSP90, RUVBL2 |
| **21** | Mechanisms of drug resistance in SCLC | 3.55E-02 | p70 S6 kinase2, HSF1 |
| **22** | DNA damage_ATM activation by DNA damage | 3.64E-02 | HSP90, MRNIP |
| **23** | Immune response_IL-2 signaling via ERK, PI3K, and PLC-gamma | 3.83E-02 | p70 S6 kinase2, p70 S6 kinases |
| **24** | DNA damage_Intra S-phase checkpoint | 4.12E-02 | MUS81, GCN5 |
| **25** | Development_Negative regulation of WNT/Beta-catenin signaling in the cytoplasm | 4.73E-02 | YAP1/TAZ, TAZ |
